# Structure of Fucoidan from Brown Seaweed *Turbinaria ornata* as Studied by Electrospray Ionization Mass Spectrometry (ESIMS) and Small Angle X-ray Scattering (SAXS) Techniques

**DOI:** 10.3390/md11072431

**Published:** 2013-07-12

**Authors:** Thuy Thi Thu Thanh, Van Thi Thanh Tran, Yoshiaki Yuguchi, Ly Minh Bui, Tai Tien Nguyen

**Affiliations:** 1 Institute of Chemistry, Vietnam Academy of Science and Technology, 18 Hoang Quoc Viet, Cau giay, Hanoi, Vietnam; E-Mail: nttai@ich.vast.ac.vn; 2 Nha Trang Institute of Technology Research and Application, Vietnam Academy of Science and Technology, 02 Hung vuong, Nha trang, Khanh hoa, Vietnam; E-Mails: vanvvlnt@yahoo.com.vn (V.T.T.T.); bminhly@nitra.vast.vn (L.M.B.); 3 Faculty of Engineering, Osaka Electro-Communication University, Hatsu-cho 18-8, Neyagawa-shi, Osaka 572-8530, Japan; E-Mail: yuguchi@isc.osakac.ac.jp

**Keywords:** fucoidan, *Turbinaria ornata*, structure, ESIMS, SAXS

## Abstract

The purpose of this study is to elucidate both the chemical and conformational structure of an unfractionated fucoidan extracted from brown seaweed *Turbinaria ornata* collected at Nha-trang bay, Vietnam. Electrospray ionization mass spectrometry (ESI-MS) was used for determining the chemical structure and small angle X-ray scattering (SAXS) provided conformational of the structure at the molecular level. The results showed that the fucoidan has a sulfate content of 25.6% and is mainly composed of fucose and galactose residues (Fuc:Gal ≈ 3:1). ESIMS analysis suggested that the fucoidan has a backbone of 3-linked α-l-Fucp residues with branches, →4)-Galp(1→ at C-4 of the fucan chain. Sulfate groups are attached mostly at C-2 and sometimes at C-4 of both fucose and galactose residues. A molecular model of the fucoidan was built based on obtained chemical structure and scattering curves estimated from molecular model and observed SAXS measurement were fitted. The results indicated that fucoidan under study has a rod-like bulky chain conformation.

## 1. Introduction

Fucoidans are sulfated polysaccharides derived from marine brown seaweed. They essentially contain fucose and sulfate groups and with some others, such as galactose, xylose, mannose and uronic acids. Fucoidan is made up of α-l-fucose units linked by (1→4) and (1→3) glycosidic bonds and sulfated at positions 2 and/or 3 and/or 4 [1,2].

Fucoidans were reported to possess various biological effects *in vitro* and *in vivo* such as anti-inflammatory, anticoagulant, antithrombotic [3,4], antiviral including anti-HIV [5,6], immunomodulatory [7], antioxidant [8], and antitumor [9].

Polysaccharides are known to reveal the biological functions by forming a specific conformation. For example, branched poly-β(1→3)-d-Glucan has a strong anti-tumor activity, which may be associated with its specific chain conformation [10], while, curdlan, a linear poly-β(1→3)-d-Glucan, has no anti-tumor activity although it assumes a triple-stranded helical conformation, but by sulfation, curdlan sulfate has anti-HIV activity [11]. Therefore, the elucidations of the molecular structure, chemical structure and conformation can expand the application of a particular polysaccharide.

Recently, many reports demonstrated that tandem electrospray ionization mass spectrometry (tandem ESIMS) was a useful technique to determine the chemical structure of anionic polysaccharides, especially fucoidan, which has a very complex structure [12,13,14]. With the development of high-resolution instrumental processes, such as scattering techniques (*i.e.*, light scattering, X-ray and neutron scattering), it is possible to study the conformation of a polysaccharide at the molecular level. Small Angle X-ray Scattering (SAXS) is a powerful technique that can provide additional structural information of high-resolution structures, and determine the conformation of molecule in solution [15,16].

*Turbinaria ornata* is categorized in the class Phaeophyceae, order Fucales, family Sargassaceae, and genus Turbinaria. *Turbinaria ornata* is distributed worldwide in subtropical and tropical regions. Chattopadhyay *et al.* [17] reported that fucoidan extracted from *Turbinaria conoides* was highly branched structure and exhibited high antioxidant ability. However, the fucoidan isolated from the Turbinaria species is still poorly investigated in contrast to other fucoidans.

Vietnam has a coastline of about 3200 km with the climate varying from subtropical in the northern part to tropical in the southern part of the country, very suitable for different seaweed species to grow. The total number of seaweed species along the coast was estimated to be nearly 650, including about 230 Rhodophyta, 125 Phaeophyta, 145 Chlorophyta and 75 Cyanophyta [18]. However, study on fucoidans from Vietnam brown seaweeds is very limited.

Our study aims to elucidate the structure of the fucoidan extracted from brown seaweed *Turbinaria ornata* collected at Nha-trang bay, Vietnam. Here, ESI-MS were used to determine chemical structure and SAXS was employed to elucidate conformational structure at molecular level of the fucoidan. In addition, in our work, the molecular model of the fucoidan was built based on the obtained chemical structure. Then, scattering curves estimated from the molecular model and observed SAXS measurement were compared in order to get useful information about structure of the fucoidan.

## 2. Results and Discussion

The results of yield and chemical analysis of fucoidan extracted from *Turbinaria ornata* species are summarized in Table 1.

**Table 1:** Yield and chemical analysis.

Yield (% dried seaweed)	Sulfate content (% mass)	Uronic acid (% mass)	Neutral sugar composition (% mol)				
			Fuc	Gal	Xyl	Man	Glu
2.215	25.6	7.8	30.3	9.0	trace	trace	trace

The fucoidan has high sulfate content (25.6%) and its sugar composition is mainly composed of two kinds of sugar with the molar ratio Fucose:Galactose ≈ 3:1. It is rare for unfractionated fucoidan to have such a simple sugar composition as this.

**Figure 1:**
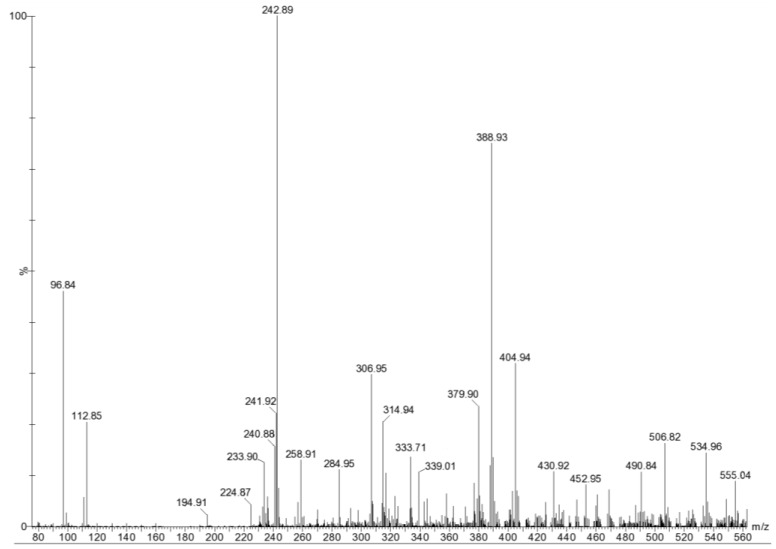
Electrospray ionization mass spectrometry (ESI-MS) of sulfated oligosaccharides derived from the hydrolysis of fucoidan of the brown seaweed *Turbinaria ornata*.

Figure 1 shows the mass spectrum of hydrolyzed fucoidan with a major signal at *m/z* 243 corresponding to the deprotonated molecule [M − H]^−^ of monosulfated fucose [FucSO_3_Na − Na]^−^. Ions at *m/z* 97 and 225 were assigned for desulfation and dehydration of monosulfated fucose, respectively. Ions at *m/z* 389 and 371 came from monosulfated difucose [Fuc_2_SO_3_Na − Na]^−^ and its dehydration, respectively. Signals at *m/z* 491 and 234 corresponded to disulfated difucose [Fuc_2_(SO_3_Na)_2_ − Na]^−^ and its doubly charged ion [Fuc_2_(SO_3_Na)_2_ − 2Na]^2−^, respectively. Signal at *m/z* 307 assigned to doubly charged ion [M − 2H]^2−^ of disulfated trifucose [Fuc_3_(SO_3_Na)_2_ − 2Na]^2−^. A minor signal at *m/z* 535 corresponds to anion [M − H]^−^ of a monosulfated trifucose [Fuc_3_SO_3_Na − Na]^−^. This spectrum also exhibits some other minor signals: [M − 3H]^3−^ of pentasulfated tetrafucose [Fuc_4_(SO_3_Na)_5_ − 3Na]^3−^ at *m/z* 333, [M − 2H]^2−^ of disulfated pentafucose [Fuc_5_(SO_3_Na)_2_ − 2Na]^2−^at *m/z* 453 and *m/z* 380 of [Fuc_4_(SO_3_Na)_2_ − 2Na]^2−^. Two signals at *m/z* 405 and 507 could arise from [FucGal(SO_3_Na) − Na]^−^ and [FucGal(SO_3_Na)_2_ − Na]^−^, respectively. The signal at *m/z* 339 assigned to [FucGlcA − Na]^−^ indicates the presence of glucuronic acid (GlcA). Some sodium adducts [M − 3H + Na]^2−^ of trisulfated difucose at *m/z* 285 and [M − 3H + Na]^2−^ of trisulfated tetrafucose at *m/z* 431 also appeared in the spectra. The results indicated that under the hydrolysis condition, the hydrolyzate was found to contain a set of fucooligosaccharides with DP 2–5 and 1–5 sulfate groups per molecule. Fragmentation of mono- and oligosaccharides are shown in Table 2.

**Table 2:** Fragmentation and proposed structure of some oligosaccharides.

*m/z*	composition	Proposed structure
96	HSO_4_^−^	
225	[FucSO_3_Na − Na − H_2_O]^−^	
234	[Fuc_2_(SO_3_Na)_2_ − 2Na]^2−^	
243	[FucSO_3_Na − Na]^−^	Fuc2S, Fuc4S
259	[GalSO_3_Na − Na]^−^	
285	[Fuc_2_(SO_3_Na)_3_ − 3Na + Na]^2−^	
307	[Fuc_3_(SO_3_Na)_2_ − 2Na]^2−^	
371	[Fuc_2_SO_3_Na − Na − H_2_O]^−^	
333	[Fuc_4_(SO_3_Na)_5_ − 3Na]^3−^	
339	[FucGlcA − Na]^−^	
380	[Fuc_4_(SO_3_Na)_2_ − 2Na]^2−^	
389	[Fuc_2_SO_3_Na − Na]^−^	Fuc2S-(1→3)Fuc Fuc4S-(1→3)Fuc Fuc(1→3)-Fuc4S Fuc(1→4)-Fuc2S
405	[FucGal(SO_3_Na) − Na]^−^	Gal(1→4)Fuc2S Fuc(1→4)Gal2S Fuc2S(1→4)Gal Gal4S(1→4)Fuc
431	[Fuc_4_(SO_3_Na)_3_ − 3Na + Na]^2−^	
453	[Fuc_5_(SO_3_Na)_2_ − 2Na]^2−^	
491	[Fuc_2_(SO_3_Na)_2_ − Na]^−^	Fuc2S(1→3)Fuc4S Fuc4S(1→3)Fuc2S
507	[FucGal(SO_3_Na)_2_ − Na]^−^	
535	[Fuc_3_SO_3_Na − Na]^−^	

**Figure 2:**
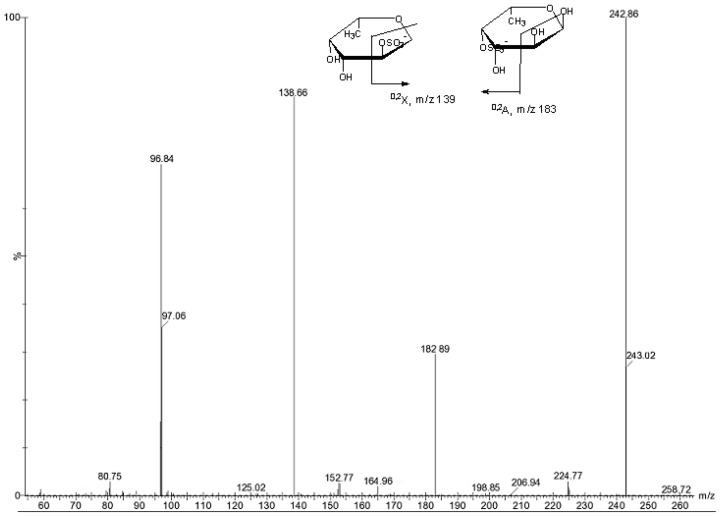
Negative ESIMS/MS of the ion [FucSO_3_Na−Na]^−^ at *m/z* 243.

The fragmentation of the most abundant [FucSO_3_Na − Na]^−^ ion at *m/z* 243 (Figure 2) led to the loss of sulfate detected at *m/z* 97. Berangere Tissot *et al*. [19] reported the effect of the sulfate position on the fragmentation pattern. Signals from C-4 (*m/z* 183) and C-2 (*m/z* 139) sulfation of α-l-Fucp residues were detected. The fragment ions at *m/z* 183 and *m/z* 139 were assigned to the fragment ions ^0,2^*A* and ^0,2^*X*, respectively. The ion at *m/z* 139 was the major fragment indicating that fucose residues of the fucoidan are mainly sulfated at position 2.

**Figure 3:**
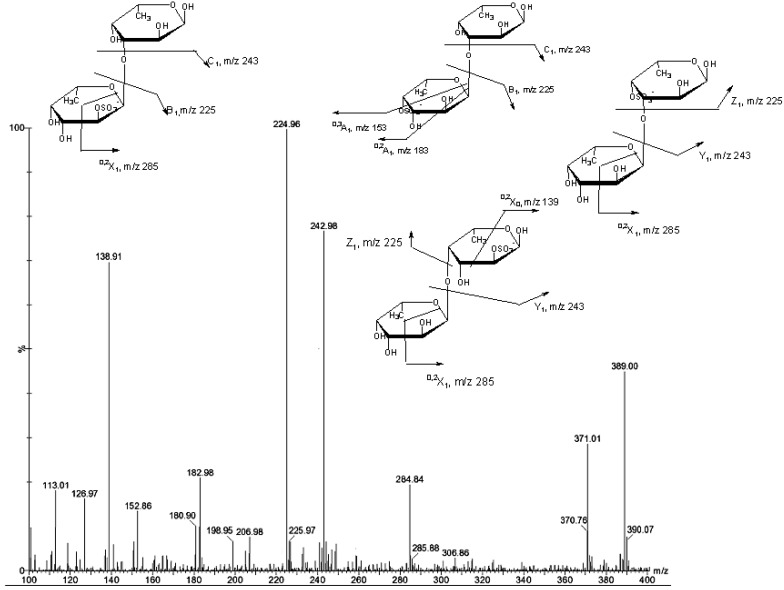
Negative ESIMS/MS of the ion [Fuc_2_SO_3_Na−Na]^−^ at *m/z* 389.

The fragmentation pattern of [Fuc_2_SO_3_Na − Na]-ion at *m/z* 389 (Figure 3) indicated that the Y_1_ and C_1_ ions at *m/z* 243 from glycosidic bond breaking had strong intensity. The spectra contained an intensive ion at *m/z* 225 (B-type) which indicates sulfation at C-2 of non-reducing fucose residues. The ^0,2^*A*_1_ and ^0,3^*A*_1_ ions at *m/z* 183 and 153 were arisen from cross-ring cleavage of the α-l-Fucp-4-OSO_3_-residues. To interpret the spectra, we used the result of Anastyuk *et al*. [20] that the formation of characteristic ^0,2^*A*-/^0,2^*X*-ions required available proton at the C-3 hydroxyl group. Our spectra lack the ^0,2^*A*_2_ ion at *m/z* 329, which were found upon ESIMS/MS of the same [Fuc_2_SO_3_Na − Na]^−^ ion containing (1→4)-linked α-l-Fucp-2-OSO_3_-residues of fucoidan from *Ascophyllum nodosum* [12,21]. This indicated that our fucoidan contained predominantly (1→3)-linked disaccharides, similar fucoidan from *Laminaria cichorioides* [22]. In contrast, the presence of a very intensive signal of ^0,2^*X*_0_ at *m/z* 139 in the spectra may be attributed to (1→4) linked difucoside sulfated at position 2 of reducing residue [α-l-Fucp-(1→4)-α-l-Fucp-2-OSO_3_]. Therefore, MS/MS fragmentation of the ion at *m/z* 389 corresponded to a mixture of four monosulfated difucose (Figure 3, Table 2, *m/z* 389).

**Figure 4:**
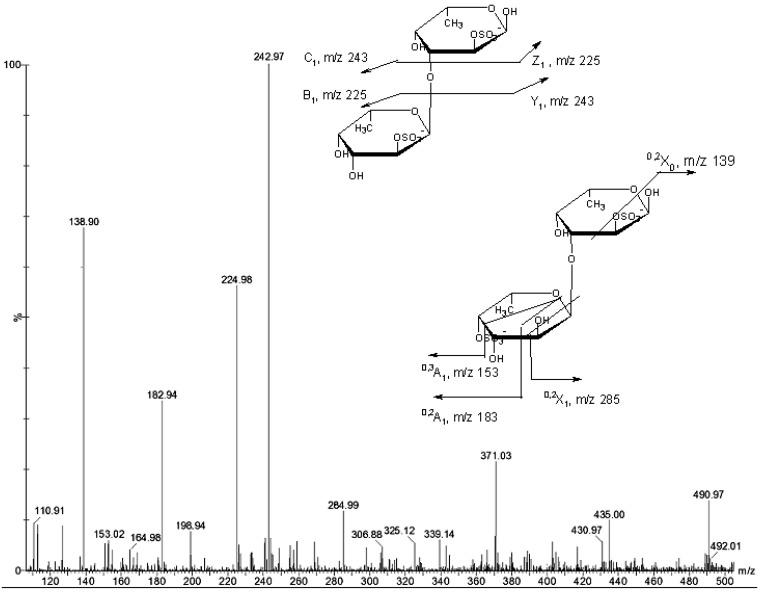
Negative ESIMS/MS of the ion [Fuc_2_(SO_3_Na)_2_−Na]^−^ at *m/z* 491.

ESIMS/MS of the ion [Fuc_2_(SO_3_Na)_2_ − Na]^−^ at *m/z* 491 (Figure 4) contain the above-mentioned Y_1_ and C_1_ ions at *m/z* 243, intensive ^0,2^*X*_0_ ion at *m/z* 139, B_1_ and Z_1_ ion at 225, ^0,2^*A*_1_ and ^0,3^*A*_1_ ions at *m/z* 183 and 153. Signals of Y-type ion and corresponding Z-type ion of disulfated fucose at *m/z* 345 and 327, respectively, could not be found. Therefore, MS/MS fragmentation of ion 491 corresponded to a mixture of two disulfated difucose (Figure 4, Table 2, *m/z* 491).

The spectral features of both 389 and 491 ions lack of ^0,2^*A*_2_ ion at *m/z* 329 and/or 204, which were intensive in the case of ESIMS/MS of oligosaccharides from *A. nodosum* [12,20] and *F. evanescens* [14], rich of (1→4)-linked α-l-Fucp residues. Summarizing the ESIMS/MS data of mono- and difucose, the following conclusions were made: our sample has an α(1→3)-linked l-fucopyranose backbone and residues are sulfated mainly at C2 and sometimes at C4 similar fucoidan fractions from brown seaweed *Laminaria cichorioides* [22] and a galactofucan from generative brown seaweed *Costaria costata* [13].

**Figure 5:**
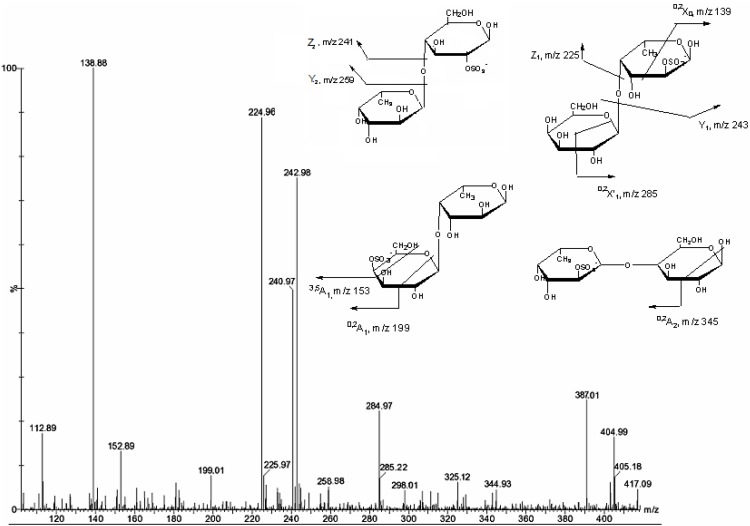
Negative ESIMS/MS of the ion [FucGal(SO_3_Na)−Na]^−^ at *m/z* 405.

ESIMS/MS of the ion [FucGalSO_3_]^−^ at *m/z* 405 (Figure 5) was very complex. The most intensive ion ^0,2^*X*_0_ at *m/z* 139 and corresponding Y_1_ ion at *m/z* 243 indicated sulfonation at *O*-2-position of the reducing end of the monosulfated α-l-Fucp residues. The spectra also had two intensive signals from sulfated at C-2 fucose residue (Z_1_ ion at *m/z* 225) and lower signal from sulfated galactose residue (Z_2_ ion at *m/z* 241), both from the reducing end. Regarding the glycosidic linkage, a previous study [23] of the fragmentation of heparin disaccharides showed that the mechanism of formation of the ^0,2^*A*_2_ ion required available hydrogen on the C-3 hydroxyl group, in order to assist the breaking of the C-2–C-3 bonds. The presence of ^0,2^*A*_2_ ion at *m/z* 345 and very high abundance of the ^0,2^*X*_0_ ion that suggested a (1→4)-type linkage [14,21]. In addition, Y-type ion from a sulfated galactose on the reducing end was found at *m/z* 259, having low intensity, and the corresponding intensive Z-type ion at *m/z* 241 suggested a structural variant →4)Gal→, in which non-reducing galactose could be sulfated at C-4 that was supported by ^0,2^*A*_1_ (*m/z* 199) and ^3,5^*A*_1_ (*m/z* 153) ion signals. We could not find any evidence for a (1→3)-type linkage of the parent ion, all structural characteristics indicated that the type of linkage between the fucose and galactose residues might be (1→4)-type linkage. Moreover, MS/MS fragmentation of ion 405 corresponded to a mixture of at least four available structures (Figure 5, Table 2, *m/z* 405).

The results of MS/MS analysis of ions 389 and 491 above suggested that (1→4)-linked units may be derived from the branching points of the molecules. We might propose that (1→4)-linked units may be derived from the branching points of the molecules. By combining with the presence of →4)-Galp(1→ of MS/MS analysis of ion 405, we may conclude that →4)-Galp(1→ was branches and attached to C-4 of fucose main chain as a branching point.

**Figure 6:**
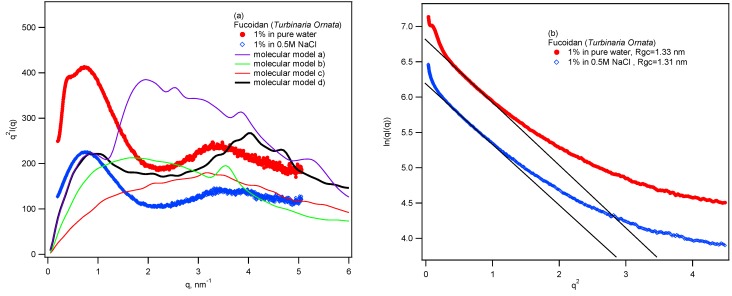
(**a**) Kratky plots; (**b**) cross-sectional Guinier plots for small angle X-ray scattering from fucoidan in water and 0.5M NaCl. The scattering curves calculated from molecular models are also shown in the figure.

Conformational structure of the fucoidan was examined by small angle X-ray scattering (SAXS). Figure 6a,b shows the Kratky and cross-sectional Guinier plots, respectively, for 1% fucoidan in water and in 0.5 M NaCl. From Kratky plots, the interference peak can be found in 0.4 nm^−1^ of *q* in the plot of sample in water, due to the repulsive electrostatic interaction indicating the present of sulfate groups. This peak is screened by adding salt as observed in the plot of sample in NaCl. From Guinier plots, the cross-sectional radius of gyration *R*gc can be estimated. The scattering from rod-like particles can be given by Guinier approximation as:

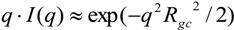
(1)

**Figure 7:**
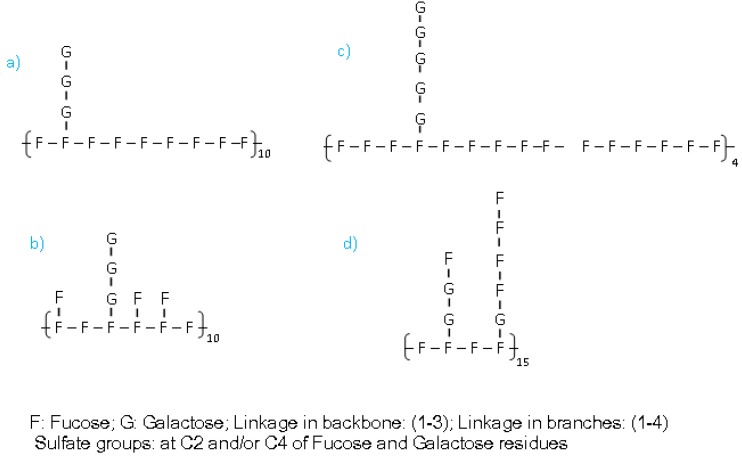
Structural units for molecular model building.

**Figure 8:**
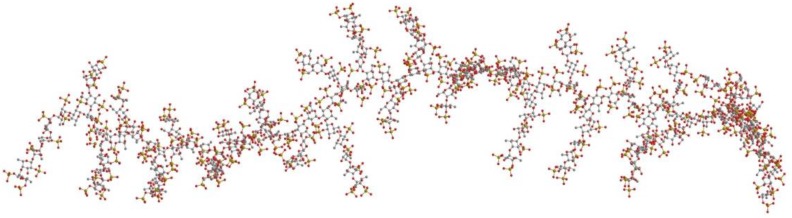
Fucoidan molecular model.

The *R*gc can be evaluated from the slope in linear region of cross-sectional Gunier plots. The *R*gc of our fucoidan estimated as 1.3 nm suggesting a bulky conformation of the fucoidan. Chemically structural determination of fucoidan is very difficult since it has a complex structure. The SAXS method provides information of local structure of the macromolecule, therefore we aim to use SAXS data and molecular model for a better understanding of fucoidan structure. Although ESIMS gave the chemical structure of oligosaccharide, we built a molecular model based on obtained chemical structure in order to find an acceptable “average structure” of the fucoidan. From ESIMS, we proposed that the backbone of the fucoidan mainly composed of 1→3 linked fucose residues; branches were galactose and/or fucose residues with 1→4 type of linkages and sulfate groups were attached at C2 and C4 of both fucose and galactose residues. Sugar analysis indicated that molar ratio Fucose:Galactose ≈ 3:1. Based on the structural information we proposed some structural units (Figure 7) and built molecular models. To find the most acceptable model, the calculated scattering curves were compared with observed SAXS curves. The result indicated that molecular model (Figure 8) built based on unit d fitted quite well with experimental curves (Figure 6a). This result can give additional information about the structure of the fucoidan, namely that our fucoidan has a very bulky structure with large branches, which are composed of both galactose and fucose residues.

## 3. Experimental Section

### 3.1. Seaweed Collection

*Turbinaria ornata* species was collected at Nha-trang bay, Vietnam in April, 2008 and identified by Dr. Le Nhu Hau (Nha-trang Institute of applied science and technology, Vietnam). A voucher specimen is deposited in Nha-trang Institute of applied science and technology. The collected seaweed was washed with tap water in order to remove salt, epiphytes, and sand attached to the surface of the samples and then dried by air in the shade. The dried seaweed was crushed and grounded into a powder form, passed through a 40-mesh sieve and stored at room temperature.

### 3.2. Extraction and Purification of Fucoidan

The extraction followed the method of Bilan *et al.* [24]. 200 g of dried seaweed was treated at room temperature with a 4:2:1 MeOH–CHCl_3_-water mixture to remove colored matter, filtered and vacuum dried to get defatted algal biomass. This material was extracted with 2% aqueous CaCl_2_ solution under mechanical stirring at 85 °C for 8 h. An aqueous hexadecyltrimethylammonium bromide solution (10%) was added to extract. The precipitate formed was centrifuged, washed with water, stirred with 20% ethanolic NaI solution for 2–3 days at room temperature, washed with ethanol, and dissolved in water. The solution was dialyzed. Fucoidan was concentrated and recovered as sodium type by freeze-drying. The amount of fucoidan obtained was 4.43 g. The yield of fucoidan was 2.215% calculated based on the dried seaweed weight. 

### 3.3. Chemical Analysis

Neutral monosaccharide compositions were elucidated by the method of Bilan *et al*. [24]. Alditol acetate derivative was prepared by hydrolysis of fucoidan sample in 2 M CF_3_COOH (TFA), 8 h at 100 °C and analyzed by 17AAFW Shimadzu GC-FID.

Uronic acid content was determined by following the carbazole method [25] using d-glucuronic acid as a standard. Interference from hexoses in this assay was determined by use of controls containing the same ratio of component sugars as found in fucoidan. Differences in the absorption characteristics of products derived from uronic acid and hexoses were used to determine the final uronic acid content.

Sulfate content was estimated using gelatin/BaCl_2_ method [26] after hydrolysis of fucoidan in 2 M TFA as described above.

### 3.4. Partial Acid Hydrolysis

Partial acid hydrolysis of the fucoidan was carried out using trifluoroacetic acid (0.75 M, 1 h, 60 °C), the solution was evaporated several times with methanol.

### 3.5. ESI-MS

ESIMS experiments were performed on a Xevo TQ MS, Waters-USA. The analyses were carried out in negative mode. Dried products of partial acid hydrolysis of fucoidan were dissolved in 1:1 MeOH-water and introduced into the mass spectrometer. Nitrogen gas was used as a nebulizer gas at 30.00 psi with a flow rate of 650 L/h and kept at 180 °C.

### 3.6. SAXS Measurement

The SAXS was observed with the small-angle X-ray scattering equipment for solution (SAXES) installed at BL-10C section of the Photon Factory, Tsukuba, Japan, from the aqueous solutions of fucoidan with or without salt at room temperature. An incident X-ray beam was monochromatized to λ = 0.149 nm and focused to the position of the detector with a bent focusing mirror. The scattered X-ray was detected by Imaging Plate (IP) positioned at a distance of about 1 m from the sample holder. The solutions were injected in a flat cell of 0.2 cm path-length made of glass with quartz windows (20 μm thick), which was placed in the cell holder kept at a predetermined temperature at least 10 min prior to the SAXS measurements. The SAXS intensities were accumulated for a total of 600 s in order to ensure enough statistical accuracy without degrading the samples by X-ray. The results were analyzed conventionally by plotting *q*^2^I(*q*) against *q* (the Kratky plots) and ln(*q*I(*q*)) against *q*^2^ (the cross-sectional Guinier plots), here I(*q*) is scattering intensity and *q* is the magnitude of scattering defined by (4πλ)sin(*θ*) with λ the wavelength of incident beam, 2*θ* scattering angle. From Guinier plots, the cross-sectional radius of gyration *R*gc can be estimated and the form of molecule in solution can be determined [15].

## 4. Conclusions

A fucoidan isolated from the brown seaweed *Turbinaria ornata* had high sulfate content and had very simple monosaccharide composition containing mainly fucose and galactose with ratio Fuc:Gal ≈ 3:1. It was classified as galactofucan. Galactofucan from *Turbinaria ornata* was shown to have a backbone of 3-linked α-l-Fucp residues, which could be sulfated at C-2 (mainly) and C-4 (partly). Sulfates also were found at C-2 (mainly) or at C-4 (partly) of →4)-Galp(1→ chains, that were attached at C-4 of a backbone as branching points. The structure of our fucoidan is similar to the main fraction of fucoidan from brown seaweed *Hizikia fusiformis* [27]. Our fucoidan has a rod-like bulky chain conformation in solution. It is the first time a combination of ESIMS, SAXS and molecular simulation has been done for structural determination of fucoidan. Although more studies are needed to obtain a fine structure of the fucoidan, this combination promised a useful way to solve the difficulty of the structural determination of fucoidan.
